# Neonatal Adverse Outcomes in Métis Children in Alberta, Canada: A Retrospective Cohort Study

**DOI:** 10.1111/ppe.70166

**Published:** 2026-06-25

**Authors:** Jesus Serrano‐Lomelin, Reagan Bartel, Ashton Anderson, Kelsey Bradburn, Susan Crawford, Jeffrey A. Bakal, Amy Colquhoun, Anne Hicks, Matthew Hicks, Manoj Kumar, Rhonda J. Rosychuk, Alvaro Osornio‐Vargas, Radha Chari, Maria B. Ospina

**Affiliations:** ^1^ Department of Public Health Sciences, Faculty of Health Sciences Queen's University Kingston Ontario Canada; ^2^ Otipemisiwak Métis Government of the Métis Nation Within Alberta (MNA) Edmonton Alberta Canada; ^3^ Alberta Health Services Provincial Research Data Services Edmonton Alberta Canada; ^4^ Health Analytics, Primary and Preventative Health Services Edmonton Alberta Canada; ^5^ Department of Pediatrics, Faculty of Medicine & Dentistry University of Alberta Edmonton Alberta Canada; ^6^ Department of Obstetrics and Gynecology, Faculty of Medicine and Dentistry University of Alberta Edmonton Alberta Canada

**Keywords:** children's health, Indigenous populations, Métis children, neonatal adverse outcome indicator, neonatal health, neonatal morbidity

## Abstract

**Background:**

Composite indicators of neonatal morbidity specific to Métis populations in Canada are lacking.

**Objectives:**

To estimate the incidence of neonatal morbidity and neonatal death among Métis neonates in Alberta, and to identify maternal and neonatal factors associated with neonatal morbidity.

**Methods:**

Population‐based retrospective cohort study including all singleton live births to Métis mothers in Alberta between April 2006 and March 2016. Hospital discharge data were used to evaluate the Neonatal Adverse Outcome Indicator (NAOI), which comprises a set of newborn medical complications and neonatal death. We estimated overall and annual NAOI incidence proportions and overall neonatal mortality. Multilevel regression models were used to estimate adjusted risk ratios (aRRs) with 95% confidence intervals (CIs) for maternal and neonatal characteristics associated with NAOI.

**Results:**

Among 7853 neonates, the overall NAOI incidence proportion was 10.1% (95% CI 9.5, 10.8), ranging annually from 7.7% (95% CI 6.1, 9.7) to 11.9% (95% CI 8.1, 17.2). The most common conditions were respiratory conditions originating in the perinatal period (4.8%, 95% CI 4.3, 5.3) and birth trauma (3.4%, 95% CI 3.0, 3.8). The overall neonatal mortality rate was 2.9 per 1000 live births (95% CI 1.9, 4.4). Increased risk of NAOI was associated with pre‐pregnancy disease (aRR 1.27, 95% CI 1.03, 1.51), pregnancy‐related disease (aRR 1.92, 95% CI 1.65, 2.18), assisted vaginal delivery (aRR 2.52, 95% CI 2.09, 2.94), caesarean delivery (aRR 1.23, 95% CI 1.04, 1.41), male sex (aRR 1.27, 95% CI 1.09, 1.42), preterm birth (aRR 4.36, 95% CI 3.71, 5.00) and any congenital malformation (aRR 2.71, 95% CI 1.87, 3.56).

**Conclusions:**

The incidence of neonatal morbidity among Métis neonates in Alberta highlights the importance of continued surveillance using standardised indicators. Incorporating NAOI into perinatal and obstetric care may inform culturally responsive improvements in care.

## 1 Background

Neonatal mortality and morbidity are key indicators of perinatal health. Neonatal morbidity reflects early life health and aspects of perinatal care and is associated with longer‐term childhood outcomes; therefore, monitoring morbidity complements surveillance focused on preterm birth, growth restriction and mortality [1, 2]. Despite global efforts to improve neonatal health, neonatal morbidity and neonatal death remain major public health concerns [3]. In 2022, an estimated 2.3 million neonatal deaths occurred worldwide, accounting for approximately 47% of all deaths among children under 5 years of age [4]. Despite a 44% global reduction in neonatal mortality from 2000 to 2022 [4], admission rates to neonatal intensive care units have shown either an upward trend [5, 6] or remained stable over the years [7]. Tracking the incidence of neonatal morbidity and neonatal death remains essential for evaluating the quality of obstetric and paediatric care, informing public health strategies and alleviating the burden on healthcare systems [8].

Persistent disparities in adverse neonatal outcomes by race, income and immigration status raise concerns about equity [6, 9]. These disparities stem from a complex web of biological, social and structural factors, including inadequate access to prenatal care, and extend beyond the neonatal period [10]. In Canada, research shows that Indigenous peoples—including First Nations, Inuit and Métis—experience poorer birth outcomes than non‐Indigenous populations [11, 12]. However, existing research has focused predominantly on commonly studied indicators such as preterm birth, small or large for gestational age, low birthweight and Apgar scores, potentially overlooking less frequent but important conditions such as infections, birth trauma and internal haemorrhages, among others. These conditions, though rarer, impose substantial demands on healthcare resources and pose serious risks to neonatal survival. The Neonatal Adverse Outcome Indicator (NAOI) [8, 13], a composite measure that includes neonatal death and a wide range of newborn complications, has been proposed to bridge this gap. It offers a comprehensive tool for integrating neonatal morbidity monitoring into both obstetric clinical care and public health and epidemiological research.

Health research and policy development programs on Indigenous populations have largely overlooked the Métis people, failing to address their unique needs and experiences [14]. Despite comprising over 600,000 self‐identifying individuals in Canada [15], including over 130,000 Métis children [16], Métis populations remain underrepresented in health research [14, 15]. Contributing factors include limited data infrastructure (e.g., lack of Indigenous identifiers and data linkages), insufficient funding for Métis‐led research, and the absence of standardised definitions of Indigenous identity in administrative health datasets [14, 15]. This study addresses the evidence gap on neonatal morbidity and neonatal death among Métis populations by estimating the incidence of the NAOI and identifying associated maternal and neonatal factors among Métis births in Alberta, Canada.

## 2 Methods

### 2.1 Study Design and Setting

We conducted a population‐based retrospective birth cohort study using de‐identified administrative health data for Métis peoples in Alberta, a culturally diverse province in western Canada with a population of approximately 4.8 million and a universal, single‐payer health care system. Alberta has the second‐largest population of self‐identified Métis people in Canada (~127,000), representing 20.4% of the national Métis population [17].

This study is part of the *Lii Zaanfaan* (‘the children’ in Michif) research project, a cohort study examining the health and well‐being of Métis children from birth to age ten. The study protocol was developed in collaboration with the Otipemisiwak Métis Government of the Métis Nation within Alberta (MNA), which represents over 70,000 registered MNA citizens. A formal research agreement outlining roles, responsibilities and data governance was signed between the investigators and the MNA. The study adheres to the guidelines of the REporting of studies Conducted using Observational Routinely‐collected health Data (RECORD) [18], the STrengthening the Reporting of OBservational studies in Epidemiology (STROBE) [19], and follows the six principles of ethical Métis research: reciprocal relationships, respect, safe and inclusive environments, diversity, Métis relevance and context considerations [20].

### 2.2 Study Population

The cohort included all singleton live births (≥ 22 weeks' gestation) to Métis mothers that occurred in Alberta between April 2006 and March 2016, as identified in the provincial clinical perinatal registry, the Alberta Perinatal Health Program (APHP). The APHP‐linked birth cohort used for this analysis includes only singleton births; therefore, multiple‐gestation births were not eligible for inclusion.

### 2.3 Data Sources and Linkage Procedures

This study used multiple linked administrative and clinical data sources in Alberta. Maternal and birth data were obtained from the Alberta Perinatal Health Program (APHP), a validated perinatal clinical registry that captures all hospital deliveries and those attended by registered midwives in Alberta [21]. Hospital admission data were obtained from the Discharge Abstract Database (DAD) [22], which includes demographic, administrative and clinical information coded using the International Classification of Diseases codes, 10th revision, Canadian version (ICD‐10‐CA codes) [23], and the Canadian Classification of Health Interventions (CCI) [23]. Sociodemographic information was obtained from Alberta Vital Statistics (birth and death records) and the Alberta Health Care Insurance Plan registry, which maintains longitudinal records of residence and basic demographic characteristics. Maternal socioeconomic status at delivery was determined using the Pampalon Material and Social Deprivation Index [24], a validated area‐based measure derived from Canadian census data. This index includes two components: material deprivation (based on income, educational attainment and employment) and social deprivation (based on marital status, living arrangements and single parenthood), each categorised into quintiles, from 1 (least deprived) to 5 (most deprived). The MNA Identification Registry, a Métis citizenship registry maintained by the MNA independently of the health system, also contains demographic information on registered MNA citizens dating back to 2004. As of 2021, the MNA Identification Registry was the largest among Métis governing bodies, with 45,350 registered citizens [15], representing approximately 35% of Alberta's Métis population [16].

Métis pregnancies were identified through probabilistic linkage between the MNA Identification Registry and the Alberta Health Care Insurance Plan registry using name (first, middle, last), date of birth and sex. This method consistently linked over 96% of MNA citizens to provincial databases (unpublished data, Alberta Health). Once MNA citizenship was linked to individuals in the provincial population registry, deterministic linkage using maternal personal identification numbers was performed to identify corresponding birth records in the APHP. Additional deterministic linkage, also using personal health numbers, was used to integrate data from administrative health data sources, including DAD and Vital Statistics. All linkage and dataset construction were conducted by Alberta Health's Analytics and Performance Reporting Branch, the APHP and the Strategy for Patient‐Oriented Research (SPOR) Data Access Platform. The final dataset was individual‐level, de‐identified and anonymised for research purposes.

### 2.4 Outcome: Neonatal Adverse Outcome Indicator

The primary outcome was the NAOI indicator, as originally proposed by Lain et al. [8] and Knight et al. [13], which is defined as a validated composite measure used to evaluate neonatal morbidity and mortality. The NAOI comprises neonatal death and a wide set of newborn complications (e.g., birth trauma, intraventricular haemorrhage, cerebral infarction, respiratory distress, congenital pneumonia, gestational age < 32 completed weeks, very low birthweight) and clinical procedures such as resuscitation and ventilatory support, among others. These medical conditions and interventions are identified during the first 28 days of life and are associated with increased risk of mortality and hospital re‐admissions within the first year [8, 13]. From the original list of NAOI components, we excluded gestational age < 32 weeks and birthweight < 1500 g because these are strong predictors of NAOI [25]. The complete list of the ICD‐10‐CA and CCI codes used to define the NAOI is provided in Table S1.

### 2.5 Maternal and Neonatal Characteristics

Maternal demographic variables included maternal age at delivery (< 20, 20–34 and ≥ 35 years), area of residence (urban/metropolitan centre or rural/small population centre, based on population density and proximity to urban areas) [26], and area‐level material and social deprivation, derived from the Pampalon Deprivation Index [24]. Maternal postal codes at delivery were linked to the 2006 Census for births between 2006 and 2010, and to the 2016 Census for births between 2011 and 2016 to assign deprivation quintiles.

Maternal clinical characteristics included smoking and substance use during pregnancy (including alcohol consumption, defined as ≥ 3 drinks on any occasion or ≥ 1 drink per day throughout pregnancy), high pre‐pregnancy weight (recorded as ≥ 91 kg in the APHP), presence of a pre‐existing medical condition (at least one of diabetes mellitus, heart disease, hypertension or renal disease) and presence of a pregnancy‐related condition (at least one of gestational hypertension, gestational diabetes, preeclampsia, eclampsia, placenta praevia, antepartum haemorrhage or premature rupture of membranes). Prenatal care utilisation [27] was categorised as adequate, intermediate, intensive, inadequate or no care. Delivery mode was categorised as spontaneous vaginal, assisted vaginal (vacuum or forceps) or caesarean section.

Neonatal characteristics included sex (female or male), gestational age groups [25] (≤ 27, 28–31, 32–36 and ≥ 37 weeks), the presence of any congenital malformation and size for gestational age classified into small for gestational age (birthweight < 10th percentile), appropriate for gestational age (birthweight between the 10th and 90th percentiles) and large for gestational age (birthweight > 90th percentile) using a Canadian sex and gestational age birthweight standard reference [28].

### 2.6 Statistical Analysis

The incidence proportion for the NAOI indicator was calculated as the number of neonates with at least one NAOI condition divided by the total number of singleton live births. Exact numbers were not reported for cells with fewer than 10 observations in accordance with data privacy research agreements. Annual NAOI incidence proportions with 95% confidence intervals (CIs) were estimated for each birth year from 2006 to 2016, along with the cumulative incidence across the full study period. A linear trend in NAOI incidence over time was assessed using the Jonckheere–Terpstra test. The incidence of each NAOI component during the first 28 days of life was calculated using all births in the cohort (cumulative incidence). In addition, NAOI incidence proportions were estimated for NAOI components (or broader categories when needed) by gestational age group.

Neonatal mortality was defined as deaths within the first 28 days of life and expressed as a rate per 1000 singleton live births during the study period. We fit multilevel logistic regression models with robust standard errors to account for within‐mother clustering due to multiple births to the same mother during the study period. The fitted models were used to estimate marginal predicted probabilities of any NAOI condition for each maternal and neonatal characteristic, from which adjusted risk ratios (aRR) were derived. To address multicollinearity and avoid over‐adjustment, we combined highly correlated variables (e.g., smoking, alcohol and substance use during pregnancy). We used a directed acyclic graph (DAG) [29] to represent hypothesised relationships among variables (Figure S1) and to identify a minimal sufficient adjustment set for estimating confounder‐adjusted associations between maternal/neonatal characteristics and NAOI, while avoiding adjustment for colliders or variables not required for confounding control [29]. We acknowledge that this approach highlights statistical associations that may not represent causal relationships. Both crude RR and aRR with 95% CI were reported.

#### 2.6.1 Missing Data

Missing data were summarised for each variable and reported in the descriptive tables. For regression models, we conducted complete‐case analyses because missingness at individual‐level covariates was minimal (< 1%) and imputing covariates in linked administrative data would require additional assumptions about the missing‐data mechanism that were not readily verifiable.

All statistical analyses were conducted using Stata (Release 16, StataCorp LLC, College Station, TX), and the DAG was constructed using DAGitty [30].

### 2.7 Ethics Approval

Institutional ethics approval was obtained from the University of Alberta's Health Research Ethics Board (#Pro00098620) and Queen's University Health Research Ethics Board (TRAQ# 6037209).

## 3 Results

### 3.1 Characteristics of the Study Population

**FIGURE 1:**
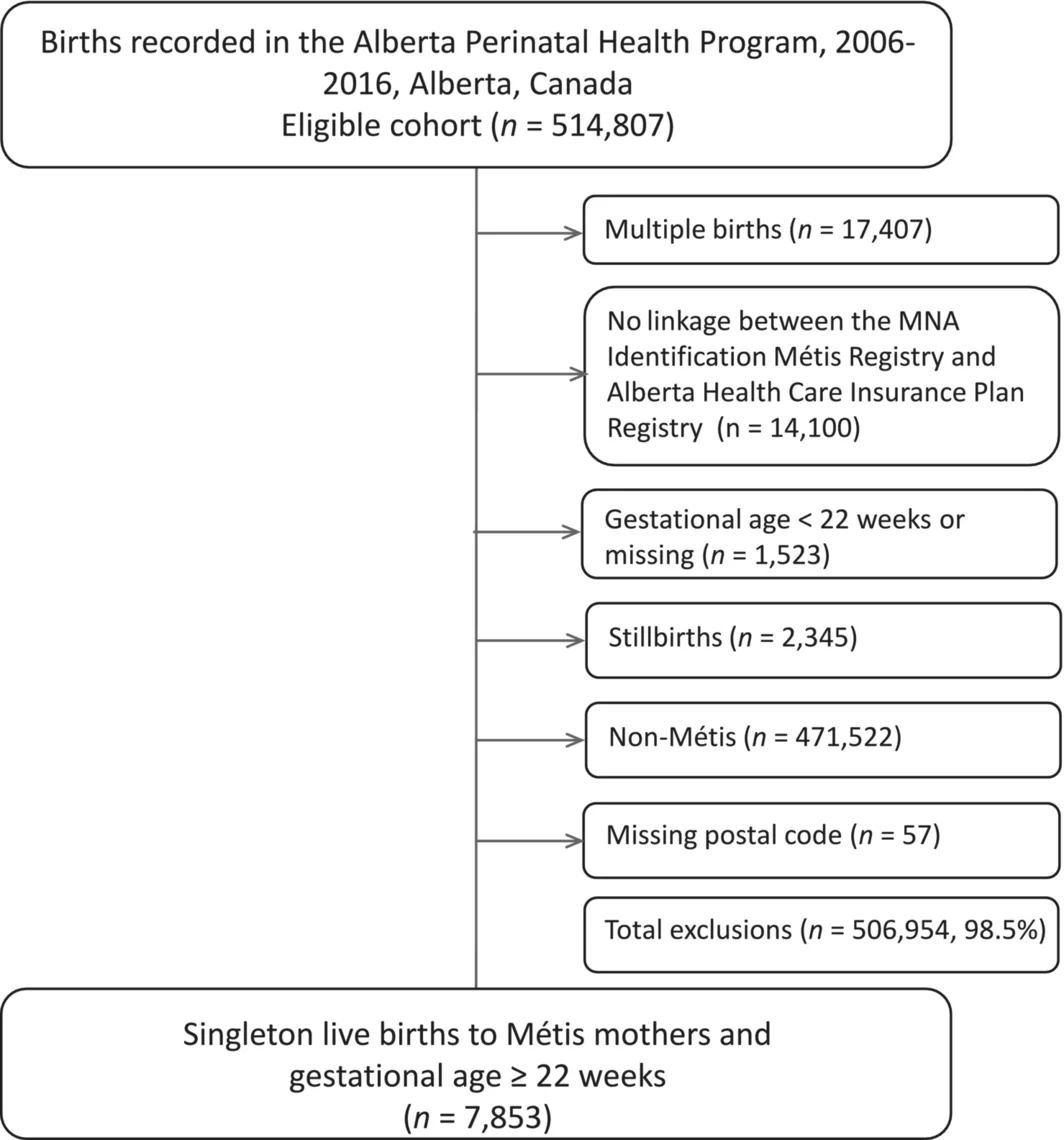
Flow diagram of cohort selection. APHP, Alberta Perinatal Health Program; MNA, Otipemisiwak Métis Government of the Métis Nation within Alberta.

The cohort included 7853 singleton live births to mothers identified as Métis (Figure 1). On average, Métis mothers had two children during the study period (mean 1.9; SD 0.9). Among them, 38% had only one child, 42% had two children, 15% had three children, and 5% had between four and seven children. Table 1 presents the maternal sociodemographic, clinical and neonatal characteristics of the cohort. Among all births, 9.5% were to mothers under 20 years of age, and 11.8% were to women aged 35 years or older. Nearly half (47.4%) of mothers resided in rural or small population centres. A higher proportion of mothers were in the two most materially deprived quintiles (50.6%) than in the two least deprived quintiles (25.7%). Similarly, 49.1% were in the two most socially deprived quintiles, compared with 25.9% in the two least deprived quintiles.

**TABLE 1:** Maternal sociodemographic, clinical and neonatal characteristics of Métis singleton live births in Alberta (April 2006–March 2016).

Characteristics of study population	*N* = 7853, *n* (%)
Maternal sociodemographic factors	
Maternal age group (years at delivery)	
< 20	748 (9.5)
20–34	6179 (78.7)
≥ 35	926 (11.8)
Area of residence at delivery	
Rural/small population centre	3723 (47.4)
Urban/metropolitan centre	4056 (51.7)
Missing	74 (0.9)
Material deprivation index	
Q_1_ (less deprived)	845 (10.8)
Q_2_	1173 (14.9)
Q_3_	1458 (18.6)
Q_4_	1740 (22.2)
Q_5_ (most deprived)	2233 (28.4)
Missing	404 (5.1)
Social deprivation index	
Q_1_ (less deprived)	892 (11.4)
Q_2_	1139 (14.5)
Q_3_	1560 (19.9)
Q_4_	1972 (25.1)
Q_5_ (most deprived)	1886 (24.0)
Missing	404 (5.1)
Maternal clinical factors	
High pre‐pregnancy weight (≥ 91 kg)	
Yes	1119 (14.3)
No	6662 (84.8)
Missing	72 (0.9)
Smoking during pregnancy	
Yes	2392 (30.5)
No	5389 (68.6)
Missing	72 (0.9)
Substance use during pregnancy	
Yes	480 (6.1)
No	7301 (93.0)
Missing	72 (0.9)
At least one pre‐existing medical condition	
Yes	865 (11.0)
No	6988 (89.0)
At least one pregnancy‐related condition	
Yes	1730 (22.0)
No	6123 (78.0)
Prenatal care utilisation	
Adequate	1626 (20.7)
Intermediate	4366 (55.6)
Intensive	146 (1.9)
Inadequate	1397 (17.8)
No care	278 (3.5)
Missing	40 (0.5)
Delivery mode	
Spontaneous vaginal	5097 (64.9)
Assisted vaginal (vacuum/forceps)	717 (9.1)
Caesarean section	2015 (25.7)
Missing	24 (0.3)
Neonatal factors	
Sex at birth	
Female	3931 (50.1)
Male	3922 (49.9)
Gestational age groups (completed weeks)	
≤ 27 weeks	30 (0.4)
28–31 weeks	44 (0.5)
32–36 weeks	493 (6.3)
≥ 37 weeks	7268 (92.8)
Size for gestational age	
Small for gestational age	550 (7.0)
Appropriate for gestational age	6290 (80.1)
Large for gestational age	997 (12.7)
Missing	16 (0.2)
Any congenital malformation	
Yes	111 (1.4)
No	7742 (98.6)

In terms of maternal clinical characteristics, 14.3% of mothers had a pre‐pregnancy weight ≥ 91 kg, 30.5% reported smoking during pregnancy, and 6.1% reported alcohol or substance use. Inadequate prenatal care was reported in 17.8% of pregnancies, while 3.5% did not receive prenatal care. Neonatal characteristics were evenly distributed by sex (49.9% male, 50.1% female). Overall, 7.2% of neonates were born preterm, while 7.0% and 12.7% were classified as small and large for gestational age, respectively. The proportion of missing data was < 1% for all individual‐level variables; however, area‐level material and social deprivation variables had 5.1% missing data.

### 3.2 NAOI Incidence

**FIGURE 2:**
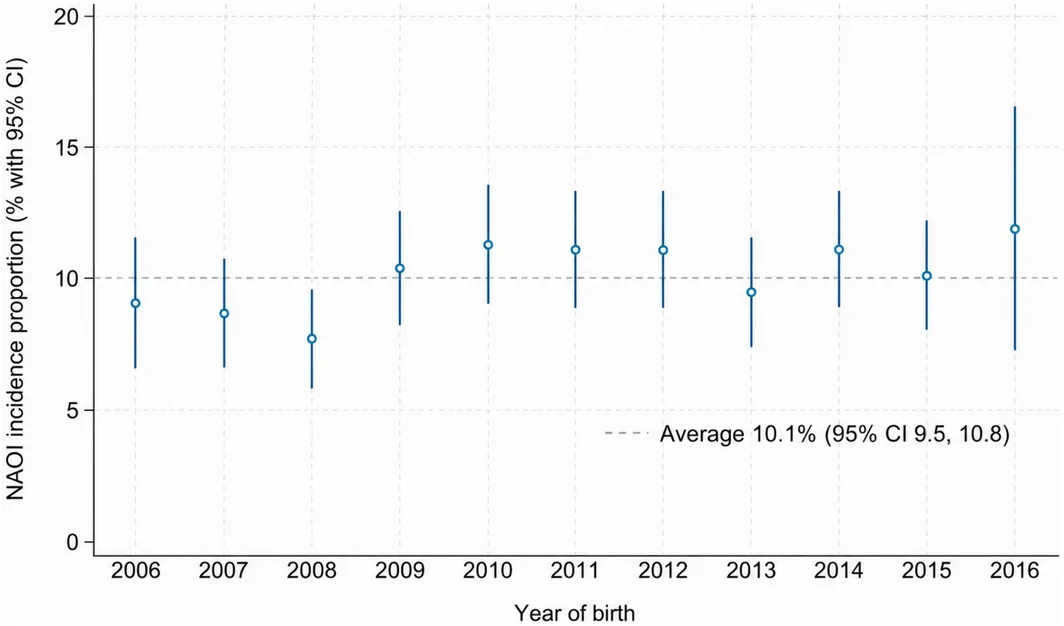
Annual incidence proportion of the Neonatal Adverse Outcome Indicator (NAOI) among Métis singleton live births in Alberta, 2006–2016. CI, confidence interval; NAOI, Neonatal Adverse Outcome Indicator.

A total of 10.1% of Métis neonates (95% CI 9.5, 10.8) experienced at least one of the conditions or clinical procedures defined by the NAOI framework. Annual NAOI incidence proportions ranged from a low of 7.7% in 2008 (95% CI 6.1, 9.7) to a high of 11.9% in 2016 (95% CI 8.1, 17.2) (Figure 2). We observed no clear linear trend in NAOI incidence over the study period.

Table 2 presents the incidence proportions for individual components of the NAOI indicator. The conditions with an incidence exceeding 1% were as follows: respiratory conditions originating in the perinatal period (4.8%, 95% CI 4.3, 5.3), birth trauma (3.4%, 95% CI 3.0, 3.8), infections specific to the perinatal period (1.4%, 95% CI 1.1, 1.7), and respiratory distress syndrome (1.3%, 95% CI 1.1, 1.6). Table 3 summarises neonatal morbidity stratified by gestational age groups for individual and broader NAOI components. All neonates born at ≤ 27 weeks' gestation experienced at least one NAOI component compared with 7.9% (95% CI 7.3, 8.6) among those born at ≥ 37 weeks. Respiratory morbidity was the most common component, ranging from 80.0% (95% CI 62.7, 90.5) in neonates born at ≤ 27 weeks to 3.3% (95% CI 2.9, 3.8) in term neonates. There were 23 neonatal deaths within the first 28 days of life, corresponding to a neonatal mortality rate of 2.9 per 1000 live births (95% CI 1.9, 4.4).

**TABLE 2:** Incidence proportion of Neonatal Adverse Outcome Indicator (NAOI) components among Métis singleton live births in Alberta, 2006–2016.

NAOI component	*n*	Total incidence proportion (%, 95% CI)
Overall NAOI (any component)	794	10.1 (9.5, 10.8)
Respiratory outcomes in the perinatal period	376	4.8 (4.3, 5.3)
Birth trauma	265	3.4 (3.0, 3.8)
Infections (specific to the perinatal period)	108	1.4 (1.1, 1.7)
Respiratory distress syndrome	106	1.3 (1.1, 1.6)
Neonatal or in‐hospital death	23	0.3 (0.2, 0.4)
Bacterial meningitis	21	0.3 (0.2, 0.4)
Chronic respiratory disease in the perinatal period	18	0.2 (0.1, 0.4)
Bronchopulmonary dysplasia	12	0.1 (0.1, 0.3)
Pneumonia (congenital pneumonia)	12	0.1 (0.1, 0.3)
Intraventricular haemorrhage	11	0.1 (0.1, 0.3)
Seizures (convulsions of newborn)	< 10	NA
Necrotizing enterocolitis of foetus or newborn	< 10	NA
Cerebral infarction	< 10	NA
Neonatal cerebral leukomalacia	< 10	NA
Clinical procedures		
Ventilatory support	78	1.0 (0.8, 1.2)
Resuscitation (intubation, chest compression)	< 10	NA
Central venous or arterial catheter	< 10	NA
Any intravenous fluid	< 10	NA
Transfusion of blood or blood products	< 10	NA

*Note:* Exact numbers for individual components with < 10 cases are suppressed.

Abbreviation: NA, not applicable due to < 10 cases.

**TABLE 3:** Incidence proportion of individual and broad NAOI components by gestational age groups among Métis singleton live births in Alberta, 2006–2016.

NAOI component	Gestational age (completed weeks)			
	[≤ 27], % (95% CI)	[28–31], % (95% CI)	[32–36], % (95% CI)	[≥ 37], % (95% CI)
Number of NAOI components				
0	0	6.8^a^	70.6 (66.6, 74.6)	92.1 (91.4, 92.7)
1	20.0^a^	25.0^a^	20.3 (16.7, 23.8)	6.9 (6.3, 7.5)
2+	80.0 (65.7, 94.3)	68.2 (54.4, 82.0)	9.1 (6.6, 11.7)	1.0 (0.8, 1.2)
Respiratory conditions: Other respiratory conditions originating in the perinatal period; respiratory distress syndrome; bronchopulmonary dysplasia; pneumonia (congenital pneumonia); chronic respiratory disease originating in the perinatal period	80 (62.7, 90.5)	90.9 (78.8, 96.4)	23.9 (20.4, 27.9)	3.3 (2.9, 3.8)
Infections (specific to the perinatal period)	30^a^	9.1^a^	2.6 (1.5, 4.5)	1.1 (0.9, 1.4)
Birth trauma	3.3^a^	4.5^a^	2.8 (1.7, 4.7)	3.4 (3.0, 3.8)
Neurological: Seizures (convulsions of newborn); cerebral infarction; bacterial meningitis; neonatal cerebral leukomalacia	0	0	0.8 (0.3, 2.0)	0.3 (0.2, 0.5)
Others: Intraventricular haemorrhage; necrotizing enterocolitis of foetus or newborn	26.7^a^	11.4^a^	0.6 (0.2, 1.8)	0.01 (0.002, 0.08)
Any clinical procedure	36.7^a^	15.9^a^	3.4 (2.2, 5.5)	0.8 (0.6, 1.0)

^a^ Excessively wide confidence interval resulted from a very small number of cases (< 10). It is considered uninformative and therefore not reported.

### 3.3 NAOI Incidence and Associated Maternal and Neonatal Factors

Table 4 presents the crude and adjusted RR for maternal and neonatal factors associated with NAOI. Increased incidence of NAOI was observed among neonates born to mothers with pre‐pregnancy medical conditions, and those with pregnancy‐related complications. Delivery‐related factors also showed elevated risk of NAOI: assisted vaginal delivery (aRR 2.52, 95% CI 2.09, 2.94) and caesarean delivery (aRR 1.23, 95% CI 1.04, 1.41). Among neonatal characteristics, male sex, preterm, small for gestational age, large for gestational age, and congenital malformations were associated with higher incidence of NAOI.

**TABLE 4:** Associations between maternal and neonatal characteristics and Neonatal Adverse Outcome Indicator (NAOI) incidence among Métis singleton live births in Alberta, 2006–2016.

Factor	NAOI %	Crude RR (95% CI)	aRR (95% CI)	Minimal sufficient adjustment set of covariates for estimating total effect
Maternal age group (years)				No other covariate
< 20	12	1.23 (0.97, 1.50)	1.23 (0.97, 1.50)	
20–34	9.7	1.00 (Reference)	1.00 (Reference)	
≥ 35	11.2	1.14 (0.92, 1.37)	1.14 (0.92, 1.37)	
Area of residence at delivery				No other covariate
Rural or small population centre	10.5	1.00 (Reference)	1.00 (Reference)	
Urban or metropolitan centre	9.7	0.92 (0.80, 1.05)	0.92 (0.80, 1.05)	
Material deprivation index				Area of residence
Q_1_ (less deprived)	10.5	1.00 (Reference)	1.00 (Reference)	
Q_2_	9.2	0.88 (0.64, 1.13)	0.88 (0.63, 1.13)	
Q_3_	10.4	1.00 (0.75, 1.24)	0.97 (0.72, 1.21)	
Q_4_	9.2	0.88 (0.66, 1.10)	0.84 (0.63, 1.05)	
Q_5_ (most deprived)	10.6	1.01 (0.78, 1.25)	0.98 (0.75, 1.21)	
Social deprivation index				Area of residence
Q_1_ (less deprived)	9.8	1.00 (Reference)	1.00 (Reference)	
Q_2_	9.1	0.96 (0.69, 1.22)	0.96 (0.69, 1.23)	
Q_3_	10.4	1.10 (0.82, 1.38)	1.11 (0.83, 1.39)	
Q_4_	9.5	0.98 (0.75, 1.23)	0.99 (0.74, 1.23)	
Q_5_ (most deprived)	10.8	1.12 (0.85, 1.40)	1.12 (0.84, 1.39)	
High pre‐pregnancy weight (≥ 91 kg)				Maternal age
Yes	11.6	1.19 (0.97, 1.40)	1.18 (0.96, 1.39)	Material/social deprivation
No	9.9	1.00 (Reference)	1.00 (Reference)	Pre‐existing medical condition
Smoking, alcohol, substance use in pregnancy				Maternal age
Yes	11	1.14 (0.98, 1.30)	1.11 (0.95, 1.27)	Area of residence
No	9.7	1.00 (Reference)	1.00 (Reference)	Material/social deprivation
At least one pre‐existing medical condition				Maternal age
Yes	12.6	1.29 (1.04, 1.53)	1.27 (1.03, 1.51)	High pre‐pregnancy weight
No	9.8	1.00 (Reference)	1.00 (Reference)	Material/social deprivation
At least one pregnancy‐related condition				Maternal age
Yes	16.4	1.93 (1.67, 2.20)	1.92 (1.65, 2.18)	Pre‐existing medical condition
No	8.4	1.00 (Reference)	1.00 (Reference)	
Prenatal care utilisation				Area of residency
Adequate	11.8	1.00 (Reference)	1.00 (Reference)	Pre‐existing medical condition
Intermediate	9.5	0.81 (0.68, 0.95)	0.86 (0.71, 1.00)	Pregnancy‐related condition
Intensive	8.2	0.70 (0.31, 1.09)	0.71 (0.31, 1.11)	
Inadequate	9.7	0.82 (0.65, 1.00)	0.87 (0.69, 1.06)	
No care	11.9	1.00 (0.66, 1.35)	1.07 (0.71, 1.44)	
Delivery mode				High pre‐pregnancy weight
Spontaneous vaginal	8.1	1.00 (Reference)	1.00 (Reference)	Pre‐existing medical condition
Assisted vaginal (vacuum/forceps)	19.7	2.41 (1.99, 2.84)	2.52 (2.09, 2.94)	Maternal age
Caesarean section	11.4	1.41 (1.19, 1.63)	1.23 (1.04, 1.41)	Maternal age
Sex at birth				No other covariate
Female	9	1.00 (Reference)	1.00 (Reference)	
Male	11.2	1.27 (1.09, 1.42)	1.27 (1.09, 1.42)	
Preterm birth				Sex at birth
Yes (< 37 weeks)	38.1	4.74 (4.10, 5.38)	4.36 (3.71, 5.00)	Maternal age
No	7.9	1.00 (Reference)	1.00 (Reference)	Pregnancy‐related condition
Birthweight for gestational age and sex				Sex at birth
Appropriate	9.5	1.00 (Reference)	1.00 (Reference)	Pregnancy‐related condition
Small for gestational age	13.1	1.38 (1.06, 1.70)	1.33 (1.03, 1.51)	High pre‐pregnancy weight
Large for gestational age	12.4	1.29 (1.05, 1.53)	1.27 (1.04, 1.51)	
Any congenital malformation				
Yes	29.7	2.96 (2.08, 3.84)	2.71 (1.87, 3.56)	Pre‐existing medical condition
No	9.8	1.00 (Reference)	1.00 (Reference)	Pregnancy‐related condition

Abbreviations: aRR, adjusted RR; RR, risk ratio.

## 4 Comment

### 4.1 Principal Findings

This study evaluated the burden of neonatal morbidity and neonatal mortality among singleton live births to Métis mothers in Alberta based on the NAOI framework. The study used individually linked data from a provincial perinatal registry, administrative health databases and the MNA Identification Registry. Findings revealed that neonatal morbidity was relatively common in this population, with respiratory disorders originating in the perinatal period and birth trauma accounting for the largest share of events. While less frequent, other outcomes such as respiratory distress syndrome and perinatal infections were also observed. Neonatal death was estimated in approximately three neonates per 1000 singleton live births.

Maternal health conditions, such as both pre‐existing and pregnancy‐related, along with delivery complications and neonatal characteristics, such as preterm birth, congenital malformations, small/large for gestational age and male sex, were associated with NAOI.

### 4.2 Strengths of the Study

This study has several important strengths. It draws on a large population‐based cohort spanning 10 years, capturing all singleton live births to Métis mothers in Alberta. Métis births were identified through linkage with the MNA Identification Registry, the largest of its kind in Canada, enhancing the specificity of cohort inclusion and advancing Indigenous data governance. The integration of administrative, clinical and registry data through robust linkage, along with the use of high‐quality hospital discharge data [22], enabled the identification of the individual components of the NAOI framework for Métis neonates. The adoption of the NAOI framework provided a comprehensive approach to assessing neonatal morbidity in relation to obstetrical practices, while incorporating a wide range of neonatal diagnoses and procedures. Finally, the study was developed in collaboration with the MNA and grounded in principles of ethical Métis research [20], ensuring that the findings are relevant, respectful and aligned with Métis community priorities.

### 4.3 Limitations of the Data

The study has limitations. The findings may not be generalizable to all Métis children in Alberta, as inclusion was limited to those whose mothers were citizens registered with the MNA. If Métis births that were not captured in the MNA registry or were not successfully linked differ systematically from linked births, NAOI incidence may be under‐ or overestimated (most plausibly underestimated if unlinked births are at higher risk), whereas non‐differential linkage failure would primarily reduce precision. In addition, because multiple gestation births were not available in the APHP‐linked cohort, findings are generalisable to singleton births, and NAOI incidence may be underestimated, given the higher morbidity risk among multifetal gestations [8]. Complete‐case analyses can introduce bias if missingness were related to NAOI and covariates; however, missingness was low for individual‐level variables (< 1%), and the direction and magnitude of any resulting bias are difficult to determine in linked administrative data. Residual confounding may persist, as unmeasured or imprecisely measured variables (e.g., smoking and substance use during pregnancy and socioeconomic status) may influence observed associations. In addition, while DAGs guided the identification of adjustment sets, complex interrelationships among explanatory variables and components of the composite NAOI may still introduce bias. Nevertheless, the identified associations are consistent with existing literature, as we describe below. Finally, the data reflect births occurring between 2006 and 2016, a timeframe determined by the data‐sharing agreement between the MNA, administrative data custodians and the research team. As a result, findings may not reflect more recent changes in clinical practice, healthcare delivery or policy affecting Métis communities. Despite this, the study provides a critical baseline for understanding neonatal morbidity among Métis populations, contributing to filling gaps in the existing knowledge base, and highlighting the need for ongoing surveillance and future research grounded in community collaboration.

### 4.4 Interpretation

Previous studies published between 2006 and 2018 have reported composite neonatal morbidity prevalence estimates ranging from 4.6% to 9.0% in general birth cohorts [31]. However, variation in the components included in morbidity indicators across studies limits direct comparability. In studies using the NAOI framework, prevalence estimates have ranged from 4.8% to 5.4% in England [8], France [32], and Canada [33]. In Canada, one study using a broader definition of neonatal morbidity, including conditions such as shoulder dystocia and other disturbances of cerebral function, reported a prevalence of 8.7% [34], while another study spanning the years 2013 to 2022 estimated an overall NAOI prevalence of 7.6% [25]. This latter study excluded infants with birthweight < 1500 g or gestational age < 32 weeks but included neonatal intestinal perforation and retinopathy of prematurity as part of the NAOI indicator [25]. In comparison, the time‐consistent NAOI incidence of approximately 10% observed in our study, despite exclusions of birthweight < 1500 g and gestational age < 32 weeks, suggests a higher burden of neonatal morbidity among Métis neonates. This finding raises concerns about potential inequities and highlights the importance of ongoing surveillance using standardised, inclusive frameworks.

Neonatal mortality in our study (2.9 per 1000 singleton live births) was slightly lower than the national estimates reported by Statistics Canada [35] for the same period (3.4–3.8 per 1000 live births) and the provincial estimates for Alberta (3.3–4.6 per 1000 live births). This difference may reflect our restriction to singleton births and differences in case definition and vital statistics registration/inclusion criteria at the peri‐viable threshold, which can affect ascertainment and comparability across data sources.

The associations observed between NAOI and maternal, clinical and neonatal factors are consistent with prior studies. Increased likelihood of neonatal morbidity has been previously reported among neonates from pregnancies complicated by pre‐existing cardiovascular conditions [33], diabetes (pre‐gestational or gestational) [34, 36], hypertensive disorders, preeclampsia and antepartum haemorrhage [36]. Delivery‐related factors such as caesarean section and assisted vaginal delivery have been associated with NAOI [33], as well as neonatal sex [8, 13, 32, 37], gestational age and size for gestational age [37], in particular large‐for‐gestational age [13, 37], which occurs frequently among Métis populations in Canada [12].

We did not observe an association between NAOI and either young mothers (< 20 years), smoking during pregnancy, or low socioeconomic status, despite prior findings reporting such associations [13, 32, 37]. However, the absence of association in our study may reflect limitations in measurement. Smoking was self‐reported, and socioeconomic status was assessed using an area‐level index, which may not fully capture individual‐level variation. Misclassification of these exposures could bias associations toward the null. This interpretation is supported by findings from a multicenter European study that also reported no significant association between individual‐level socioeconomic status and neonatal morbidity [38].

## 5 Conclusions

This study found a substantial burden of neonatal morbidity among Métis singleton births in Alberta. The consistent application of the NAOI framework enabled the identification of adverse neonatal outcomes and maternal and neonatal characteristics associated with increased risk. These findings highlight the need for continued surveillance using standardised indicators and support the development of culturally safe and responsive perinatal care that reflects the priorities and experiences of Métis communities.

## Author Contributions

Funding acquisition: M.B.O.; study design: M.B.O., R.B., A.A., K.B., S.C., J.A.B., A.C., A.H., M.H., M.K., R.J.R., A.O.‐V., R.C.; data preparation: A.C., S.C., J.A.B., J.S.‐L.; data analysis: J.S.‐L., M.B.O.; interpretation of results: all authors; draft of manuscript: J.S.‐L., M.B.O.; critical revision of the manuscript: all authors; review of the final version of the manuscript and approval for submission for publication: all authors.

## Funding

This study was funded by the Canadian Institutes of Health Research (Project Grant 427862) and through the Canada Research Chairs Program (CRC‐2023‐00153). The research was also formerly supported by the Alberta Women's Health Foundation through the Women and Children's Health Research Institute.

## Conflicts of Interest

The authors declare no conflicts of interest.

## Supporting information


**Table S1:** Diagnostic and intervention codes used to define components of the Neonatal Adverse Outcome Indicator (NAOI).

**Figure S1:** Directed acyclic graph.

## Data Availability

Data supporting this research cannot be shared publicly due to ethical and privacy concerns outlined in the Alberta *Health Information Act*. The datasets are held securely in coded form at Primary and Preventative Health Services (PPHS) and Alberta Health Services. Until May 2025 and during the time of this work, the Alberta Ministry that held these datasets was called Alberta Health (AH); as a result, AH will be the Ministry referenced herein. Alberta Health and Alberta Health Services are the legal custodians of the original data. Alberta Health and Alberta Health Services' policies and acts (e.g., the *Health Information Act* of Alberta) guarantee the security, privacy and confidentiality of patient data. The data agreement with Alberta Health and Alberta Health Services prohibits researchers from making the dataset publicly available. Access to data may be granted to those who meet pre‐specified criteria for confidential access. Data are available from Alberta Health Services Provincial Research Data Services for researchers who meet the criteria for access to confidential data. The health data underlying the results presented in the study are available from Alberta Health Services' (AHS) Health System Access (HSA): https://www.albertahealthservices.ca/research/page8579.aspx. More information at: research.administration@ahs.ca.
