# Supplementary material for: Neonatal Adverse Outcomes in Métis Children in Alberta, Canada: A Retrospective Cohort Study
Source: Paediatr Perinat Epidemiol. 2026 Jun 25;40(6):733–44. doi: 10.1111/ppe.70166 (PMC13522652; doi:10.1111/ppe.70166)
Supplement: Supplementary file 1 [file PPE-40-733-s002.docx]

**Table S1**. Diagnostic and intervention codes used to define components of the Neonatal Adverse Outcome Indicator (NAOI)

| **NAOI Component** | **Codes** |
| --- | --- |
| Diagnoses | ICD-10-CA |
| Birth trauma | P10-P15 |
| Necrotizing enterocolitis of fetus or newborn | P77 |
| Seizure (convulsions of newborn) | P90 |
| Intraventricular hemorrhage | P52.0-P52.3 |
| Cerebral infarction | I63 |
| Periventricular leukomalacia (Neonatal cerebral leukomalacia) | P91.2 |
| Hypoxic ischaemic encephalopathy | P91.6 |
| Bacterial meningitis | G00-G03 |
| Respiratory distress syndrome | P22.0 |
| Bronchopulmonary dysplasia | P27.1 |
| Chronic respiratory disease originating in the perinatal period | P27 |
| Pneumonia (Congenital pneumonia) | P23 |
| Other respiratory conditions originating in the perinatal period | P28 |
| Infection (Infections specific to the perinatal period) | P35-P39 |
| Neonatal or in-hospital death (first 28 days) ***^a,b^*** | NA |
| Clinical procedures | CCI |
| Resuscitation (intubation, chest compression) | 1GZ30* 1HZ30* 1HZ34* |
| Central venous or arterial catheter | 1IS53* |
| Any intravenous fluid | 1KX35* 1KX53* |
| Transfusion of blood or blood products | 1LZ19* |
| Therapeutic hypothermia | 1ZX06* |
| Pneumothorax requiring intercostal catheter | 1GT33* |
| Ventilatory support | 1GZ31* 1GZ32* |

^a^ Reported in the Alberta Perinatal Health Program database

*^b^* Reported in the Alberta Registry Database

ICD-10-CA: International Classiﬁcation of Diseases, 10th Revision, Canada.2015.

CCI: Canadian Classiﬁcation of Health Interventions

NA: not applicable
